# Effects of global financial crisis on funding for health development in nineteen countries of the WHO African Region

**DOI:** 10.1186/1472-698X-11-4

**Published:** 2011-04-13

**Authors:** Joses M Kirigia, Benjamin M Nganda, Chris N Mwikisa, Bernardino Cardoso

**Affiliations:** 1 World Health Organization, Regional Office for Africa, B.P. 06, Brazzaville, Congo

## Abstract

**Background:**

There is ample evidence in Asia and Latin America showing that past economic crises resulted in cuts in expenditures on health, lower utilization of health services, and deterioration of child and maternal nutrition and health outcomes. Evidence on the impact of past economic crises on health sector in Africa is lacking. The objectives of this article are to present the findings of a quick survey conducted among countries of the WHO African Region to monitor the effects of global financial crisis on funding for health development; and to discuss the way forward.

**Methods:**

This is a descriptive study. A questionnaire was prepared and sent by email to all the 46 Member States in the WHO African Region through the WHO Country Office for facilitation and follow up. The questionnaires were completed by directors of policy and planning in ministries of health. The data were entered and analyzed in Excel spreadsheet. The main limitations of this study were that authors did not ask whether other relevant sectors were consulted in the process of completing the survey questionnaire; and that the overall response rate was low.

**Results:**

The main findings were as follows: the response rate was 41.3% (19/46 countries); 36.8% (7/19) indicated they had been notified by the Ministry of Finance that the budget for health would be cut; 15.8% (3/19) had been notified by partners of their intention to cut health funding; 61.1% (11/18) indicated that the prices of medicines had increased recently; 83.3% (15/18) indicated that the prices of basic food stuffs had increased recently; 38.8% (7/18) indicated that their local currency had been devalued against the US dollar; 47.1% (8/17) affirmed that the levels of unemployment had increased since the onset of global financial crisis; and 64.7% (11/17) indicated that the ministry of health had taken some measures already, either in reaction to the global financing crisis, or in anticipation.

**Conclusion:**

A rapid assessment, like the one reported in this article, of the effects of the global financial crisis on a few variables, is important to alert the Ministry of Health on the looming danger of cuts in health funding from domestic and external sources. However, it is even more important for national governments to monitor the effects of the economic crisis and the policy responses on the social determinants of health, health inputs, health system outputs and health system outcomes, e.g. health.

## Background

### Overview of macroeconomic effects

Since 2008, the severe reduction in global demand for commodities, goods and services as a result of the liquidity crisis and the loss of trust in the financial sector in the United States of America and Europe has considerably slowed down the global economy. According to the International Monetary Fund (IMF), world output was expected to contract by 1.4% in 2009 and to gradually pick up in 2010 to reach a growth rate of 2.5% [1]. Although Africa registered a real average gross domestic product (GDP) growth rate of above 5% between 2000 and 2008 [2], the growth rate declined to 2.8% in 2009 [3].

The total nominal GDP (i.e. unadjusted for inflation or deflation, and measured in current year dollars) in the African Region shrunk by US$ 94.48 billion (8.6%) between 2008 and 2009; 27 countries recorded a decrease in GDP, varying widely from US$ 0.007 billion to US$ 15 billion (Table 1). Similarly, GDP per capita decreased by between US$ 6 and US$ 6183 in 31 countries [4] (Table 2). Table 2 shows that the decrease in GDP per capita was largest among oil producing countries such as Algeria, Angola, Equatorial Guinea, Gabon and Nigeria. Per capita GDP in Botswana, whose major source of foreign exchange is diamonds, declined by US$1,559. Seychelles, a tourism dependent economy, lost US$1,957 per capita.

**Table 1:** Changes in gross domestic product in the African Region (US$ billions, current prices)

	GDP		
Country	Year 2008	Year 2009	Change
Algeria	159.669	134.797	-24.872
Angola	84.945	69.708	-15.237
Benin	6.712	6.401	-0.311
Botswana	13.461	10.808	-2.653
Burkina Faso	8.116	7.780	-0.336
Burundi	1.097	1.410	0.313
Cameroon	23.732	21.820	-1.912
Cape Verde	1.744	1.755	0.011
Central African Republic	1.997	1.983	-0.014
Chad	8.400	6.974	-1.426
Comoros	0.532	0.525	-0.007
Democratic Republic of Congo	11.629	11.104	-0.525
Congo, Republic of	10.774	8.632	-2.142
Côte d'Ivoire	23.508	22.909	-0.599
Equatorial Guinea	18.525	11.175	-7.35
Eritrea	1.479	1.694	0.215
Ethiopia	26.393	33.920	7.527
Gabon	14.535	10.936	-3.599
Gambia, The	0.810	0.726	-0.084
Ghana	16.654	14.761	-1.893
Guinea	4.517	4.436	-0.081
Guinea-Bissau	0.461	0.438	-0.023
Kenya	29.564	30.212	0.648
Lesotho	1.618	1.624	0.006
Liberia	0.850	0.868	0.018
Madagascar	9.463	8.974	-0.489
Malawi	4.268	4.909	0.641
Mali	8.774	8.757	-0.017
Mauritania	3.161	3.241	0.08
Mauritius	8.738	9.156	0.418
Mozambique	9.897	9.654	-0.243
Namibia	8.835	9.039	0.204
Niger	5.382	5.323	-0.059
Nigeria	207.116	165.437	-41.679
Rwanda	4.459	5.011	0.552
Sao Tome and Principe	0.175	0.189	0.014
Senegal	13.350	12.610	-0.74
Seychelles	0.822	0.656	-0.166
Sierra Leone	1.953	2.064	0.111
South Africa	276.764	277.379	0.615
Swaziland	2.840	2.929	0.089
Tanzania	20.668	22.159	1.491
Togo	2.890	2.771	-0.119
Uganda	14.565	15.658	1.093
Zambia	14.654	12.293	-2.361
Zimbabwe	3.145	3.556	0.411
**TOTAL**	**1093.641**	**999.161**	**-94.480**

Source: IMF [4].

**Table 2:** Gross domestic product per capita in Member States of the WHO African Region (US$, current prices)

Country	Per capita GDP in 2008	Per capita GDP in 2009	Change
Algeria	4,588	3,816	-772
Angola	5,054	4,027	-1,027
Benin	828	765	-63
Botswana	7,554	5,995	-1,559
Burkina Faso	578	542	-36
Burundi	138	174	36
Cameroon	1,224	1,095	-129
Cape Verde	3,464	3,419	-45
Central African Republic	459	446	-12
Chad	863	699	-164
Comoros	816	788	-28
Democratic Republic of Congo	185	171	-13
Congo, Republic of	2,952	2,298	-654
Côte d'Ivoire	1,132	1,071	-61
Equatorial Guinea	14,941	8,759	-6,183
Eritrea	295	328	33
Ethiopia	333	418	84
Gabon	9,998	7,414	-2,583
Gambia, The	497	434	-62
Ghana	739	639	-100
Guinea	439	418	-21
Guinea-Bissau	264	244	-20
Kenya	838	842	4
Lesotho	660	651	-9
Liberia	216	210	-6
Madagascar	468	432	-36
Malawi	313	352	40
Mali	657	641	-16
Mauritania	1,042	1,044	1
Mauritius	6,872	7,146	274
Mozambique	477	456	-21
Namibia	4,278	4,341	63
Niger	391	375	-16
Nigeria	1,401	1,089	-312
Rwanda	465	512	47
São Tomé and Príncipe	1,094	1,160	66
Senegal	1,066	984	-83
Seychelles	9,640	7,683	-1,957
Sierra Leone	332	342	10
South Africa	5,685	5,635	-49
Swaziland	2,778	2,854	76
Tanzania	520	547	27
Togo	436	408	-28
Uganda	455	472	17
Zambia	1,248	1,027	-221
Zimbabwe	268	303	35

Source: IMF [4].

The contraction of GDP has been attributed to declines in private household expenditures, business enterprise purchases, government revenues, and exports of goods (e.g. crude oil, minerals, agricultural products) and services (e.g. tourism) [3,5]. Other impacts of the crisis include falls in foreign exchange rates [3], reduced foreign direct investment, decreased official development assistance (ODA) and other donor support, increased interest rates and risk premiums, and reduced remittances from abroad [6].

### Expected effects on health sector funding

There is ample evidence from Asia [7,8] and Latin America [9] showing that economic and financial crises resulted in cuts in expenditures on health, lower utilization of health services, and deterioration of child and maternal nutrition and health outcomes.

Owing to reductions in the size and growth of GDP, unless protected, the per capita spending on health and other social sectors is likely to decrease. Evidence from previous Latin America economic crises shows that governments tend to decrease social expenditures during times of economic recession [8,9]. Indonesian experience indicates that the health budget tends to be especially vulnerable to reductions during times of financial and economic crisis [7]. The proportion of government health ministry budgets going to salaries (already high in many countries) tends to increase as capital spending and other operating expenditures declines. Reductions in maintenance, medicines or other operating expenditures related to disease surveillance or supervision are likely to have a more damaging and immediate effect on quality and quantity of health service delivery [7].

Decreased real per capita household spending on health, coupled with increased costs of treatment and low coverage of prepaid health schemes will lower household demand for private sector health services, with demand switching to the public sector [6]. Because the public sector is already facing reduced funding, it may not be adequately equipped to absorb any surges in demand, and the result may be a worsening in quality of care. In most countries of the African Region, publicly-funded health services were already overstretched long before the onset of the crisis.

During periods of economic crisis, poorer households are likely to suffer the most as they are unable to re-adjust and cushion their expenditures, often forcing a decline in demand for health services [6,10]. As economic activity slows down and unemployment rises, both labour and non-labour incomes tend to decline, resulting in reduced real per capita household spending on health and other social services [9]. Argentinean experience demonstrated that without targeted pro-poor interventions or safety nets, the poor are disproportionately affected in terms of utilization of health services [11].

Poor households are also forced to reduce food quantity (caloric intake) and quality (dietary diversity), resulting in weight loss and severe malnutrition [12]. Children who experience short-term nutritional deprivations can suffer long-lasting effects including retarded growth, lower cognitive and learning abilities, lower educational attainment, and, consequently, lower earnings in adulthood [13,14].

Although donor countries and international financial institutions have recently made strong commitments to help, past banking crises have led to sharp declines in ODA, including health development assistance. For example, the Global Fund to Fight AIDS, Tuberculosis and Malaria is facing a financing gap of US$ 4 billion which may lead to reduced funding for programmes [15]. However, reducing ODA for health at this time could be very costly in terms of increased morbidity and premature mortality, especially for low-income African countries which are striving to achieve health-related Millennium Development Goals.

There is growing evidence in the WHO African Region of economic inefficiencies in the use of resources allocated to health facilities [16], medicines procurement, distribution systems and prescribing practices [17,18]. Other inefficiencies include misallocation of resources by across regions in countries, levels of care (investment of most public resources in tertiary and secondary hospitals instead of first level hospitals and health centres) and channelling most donor funds through vertical programmes instead of national health systems [19]. During the Asian and Latin American economic crises, some countries used donor funding to restructure and improve systems of public tax revenue collection and expenditures.

There is thus a real danger that funding for health development in the African Region might be adversely affected by the ongoing global financial crisis and thereby compromise any ongoing national and international efforts in many countries to realize the Millennium Development Goals [20]. Therefore, there is need for concerted action from governments and development partners in the WHO African Region to ensure that domestic and external funding for the health sector is not reduced.

### Overview of health sector funding in the WHO African Region

In 2008, 15 out of the 46 countries in the WHO African Region spent less than 5% of their GDP on health. Only five countries spent above 9% of their GDP on health [21]. Government expenditure on health as a percentage of total health expenditure in the Region varies widely from less than 11% to over 83.8%. Only five countries have met the Abuja target of allocating at least 15% of the government budget to health.

In most countries, private expenditures on health constitute approximately 49.5% of total health expenditure, a large proportion of which consists of household out-of pocket expenditures. In 32 countries, out-of-pocket expenditures account for more than 61% of private health expenditure.

External funding for health as a percentage of total health expenditure accounts for a substantial proportion of health expenditures in some African countries. In 2008, 23 countries (50% of countries of the WHO African Region) received between 20.3% and 63.5% of their total health funding from external sources [21].

There is lack of evidence about how past economic crises in the African Region affected health system funding including effects on inputs, service outputs and health outcomes [22]; as well as on the social determinants of health that shape people's daily lives and their differential access to money, power and resources which significantly affect health inequities both within and between countries [23].

Monitoring the effects of the financial crisis on health-sector spending in countries of the Region is a challenge because 19 countries have not undertaken even a single round of national health accounts (NHA), and most countries have not institutionalized NHA. The lack of institutionalization of NHA makes it difficult to track changes in funding from all sources as well as flows to various health system inputs, service providers and beneficiaries. The institutionalization of NHA in itself is however, not sufficient. An economic crisis can influence health outcomes through the social determinants of health, e.g. education, environment, food, housing, water and sanitation [24].

The rapid survey reported in this article was meant to avail the ministries of health in the African Region with a way to monitor the effects of global economic crisis on government and donor funding for health development, prices of medicines and basic foodstuffs, currency devaluation and unemployment. The survey was also meant to inform development of an information document entitled 'the global financial crisis: implications for the health sector in the African region' [25] for discussion at the 60^th ^Session of the WHO Regional Committee of the African Ministers of Health for the WHO African Region.

## Methods

In May 2009 a questionnaire entitled '*Continuous monitoring of the effects of global financial crisis on funding for health development*' [26] was developed at WHO (see Additional File 1). The questionnaire was consciously kept short and simple to ensure quick response. It consisted of 14 questions geared at obtaining information on whether: the Ministry of Health (MOH) had been notified by the Ministry of Finance that the budget for health would be cut; any development partner (donor) had notified the MOH that their funding commitments would be cut; there was any indication that the prices of medicines had increased recently; there was any indication that the prices of basic food stuffs had increased recently; the local currency had been devalued vis-à-vis the United States Dollar and/or the EURO; the levels of unemployment increased since the global financial crisis; the MOH had taken any policy measures already, either in reaction to the crisis, or in anticipation; and whether the respondents thought there were other measures that national authorities should take to mitigate any negative effects of the global financial crisis on funding for health.

The questionnaire was developed in English and subsequently translated into French and Portuguese. Of the 46 Member States in the WHO African Region, 21 have French as official language, 20 English and 5 Portuguese. It was sent by email to each of the 46 countries through the WHO country offices for facilitation and follow up. At the country level the questionnaire was completed by the Directors of Policy and Planning in Ministries of Health. The data was entered and analyzed in Excel spreadsheet.

The quick survey reported in this article assumed that the all directors of policy and planning have up-to-date information on the effects of global financial crisis in their countries. The assumption might hold since Directors of Policy and Planning are often economists seconded from the Ministries of Finance, Economic Development and Planning. We did not ask whether in the process of completing the questionnaire they consulted with the relevant ministries, e.g., agriculture, finance, labour.

## Results

A total of 19 (41%) of the 46 countries completed the questionnaire and returned it to the WHO Regional Office for Africa. Response rates of 50% (10/20), 23.8% (5/21) and 80% (4/5) were recorded for the English, French and Portuguese respectively. The respondent English speaking countries were Botswana, Gambia, Ghana, Malawi, Namibia, Nigeria, Seychelles, Sierra Leone, Uganda and Zimbabwe. Five French speaking countries that responded were Benin, Gabon, Guinea, Mauritania, and Rwanda. The four Portuguese speaking countries that responded were Angola, Cape Verde, Mozambique, and Sao Tome and Principe. It would have been preferable if all the countries had completed the questionnaire to give a regional picture of the effects of the global financial crisis on funding for health and coping strategies.

In total, nineteen countries responded to the question asking: '*Has the Ministry of Health been notified that the budget for health will be cut as a result of the current global financial/economic crisis? (i) YES (ii) NO (iii) Don't know.' *Of the 19 countries that responded, 37% (7/19) reported that the MOH had been notified by the Ministry of Finance (MOF) that the budget for health will be cut as a result of the current global financial/economic crisis. This meant that 63% (12/19) of the respondent countries did not expect reductions in their health budgets due to the global financial crisis.

Eight countries responded to the question asking "*If yes in question (a), do they know if, at the same time, the share of total government spending going to health will (i) Increase (ii) Fall (iii) Stay the same (iv) Do not know." *Only Rwanda indicated that it expected the share of total government spending going to health to increase. Angola, Ghana and Seychelles expected the share of total government spending on health to fall. Benin and Nigeria expected government spending on health to stay the same. Guinea and Mozambique indicated that they did not know whether the share of total government spending going to health would increase, fall or stay the same.

All of the 19 countries responded to the question: "*Has any development Partner (donor) notified the Ministry of Health that their funding commitments will be cut as a result of the current global financial/economic crisis? (i) Yes (ii) No (iii) Don't know." *Three (Guinea, Mozambique and Zimbabwe) indicated that the MOH had been notified by partners of their intention to cut back their funding commitments. The remaining 16 countries indicated that they had not received any notification from partners of intention to reduce their funding commitments due to the current global financial crisis. Guinea had received notification from European Union and Mozambique from some unnamed donors.

Eighteen countries answered the question asking: "*Is there any indication that the prices of medicines have increased recently? (i) Yes (ii) No (iii) Do not know. If Yes in preceding question, is there any information available about how much it has increased?" *Eleven (61%) indicated that the prices of medicines had increased recently; five (28%) reported there were no indications of increases in prices of medicines; and two (11%) said they did not know. When asked whether there was any information available about by how much prices of medicines had increased, Malawi and Sierra Leone reported 5%; Zimbabwe reported 5% to 15%; Seychelles reported 15%; Angola and Namibia reported a 25% to 30% increase.

Eighteen countries responded to the question: "*Is there any indication that the prices of basic food stuffs have increased recently: (i) Yes, (ii) No, (iii) Do not know?" *Fifteen (83%) responses were affirmative. Gambia, Malawi and Namibia indicated that prices of basic food stuffs had not increased.

Eighteen countries answered the question: "*Has the local currency been devalued since the global financial crisis against the United States of America Dollar (US$)? (i) Yes, (ii) No, (iii) Do not know. If yes, by what percentage?" *Only 7 (39%) of the respondent countries indicated that their local currency had been devalued against the US dollar. Seychelles and Zimbabwe reported devaluations of up to 100%. Angola and Sierra Leone reported devaluation of 25% and 10% respectively. Ghana, Guinea and Nigeria reported devaluations of between 0.84% and 2.5%. The remaining eleven countries reported that there had been no devaluation of their local currency.

Seventeen countries addressed the question: "*Have the levels of unemployment increased since the global financial crisis? (i) Yes, (ii) No, (iii) Do not know. If yes, what are the reasons for increasing unemployment?" *Forty-seven per cent (8/17) answered yes; 12% (2/17) answered no; and 41% (7/17) did not know. Those that answered affirmatively were asked to state the reasons for the increased unemployment. Some of the reasons given for increased unemployment included return of emigrants from Europe and America; low absorptive capacity of the oil dependent economies; introduction of the macroeconomic framework programme which entailed downsizing of the public sector; erosion of corporate sector profit margins; closure of some of the business enterprises due to economic down-turn; and increased costs of production vis-à-vis revenues. Zimbabwe indicated that the unemployment due to already existing domestic economic crisis was worsened by the global financial crisis.

Seventeen countries responded to the question asking: *"Has the Ministry of Health taken any policy measures already, either in reaction to the crisis, or in anticipation? (i) Yes, (ii) No." *Sixty-five per cent (11/17) indicated that the ministry of health had already taken some measures, either in reaction to the crisis, or in anticipation of it. The rest (35% - 6/17) of the countries indicated that their MOH had not taken any measures to mitigate the negative effects of the global financial crisis.

**Figure 1:**
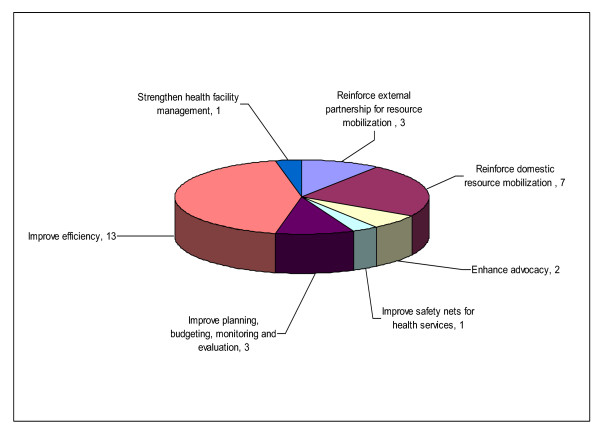
**Measures that ministries of health have undertaken to mitigate negative effects of the global financial crisis on funding for health**.

Countries that responded to the preceding question were asked to indicate what measures the Ministry of Health had already taken to mitigate any negative effects of possible reduction in funding for health. Twelve countries (63.2%) responded to this question. Figure 1 shows the broad strategies that the ministries of health have undertaken. The four strategies most frequently cited by countries were improving efficiency in allocation and use of resources (66.7%); reinforcement of domestic resource mobilization (23.1%); improvement in planning, budgeting, monitoring and evaluation (33.3%); and reinforcement of external partnership for resource mobilization (16.7%). Table 3 presents the broad strategies and specific policy measures that the ministries of health have already undertaken to mitigate any negative effects of possible reduction in funding for health.

**Table 3:** Measures that the ministry of health has taken to mitigate

Strategies	Measures
Reinforce external partnership for resource mobilization	Establishment of a funding office in MOH to identify and attract partners
Reinforce domestic resource mobilization	(a) Introduction of fees for certain services, e.g. ambulance services, mortuary service, etc.; (b) charges for certain non essential medicines
Enhance advocacy	Advocacy for fiscal policy reform to provide more financial assistance to health facilities, e.g. the earmarked tax revenues
Improve safety nets for health services	Free anti-retroviral medicines
Improve planning, budgeting, monitoring and evaluation	(a) Creation of budget line for vaccines; (b) government-wide integrated action taken to develop a plan to mitigate negative effects of the crisis; (c) development of Medium Term health expenditure framework; (c) avoidance of expenditures outside the national health strategic plan.
Improve efficiency	(a) Implemented strategies for better use of resources; (b) started importing cheaper medical supplies from Asian countries; (c) reduction in the Ministry's redundant labour force through Voluntary Departure Scheme, e.g. in Seychelles; (d) discussions underway on how to improve productivity of labour since it accounts for the largest expenditure in the sector; (e) re-prioritization of funding for sector priorities, e.g. Rwanda; (f) measures to related to cutbacks (restriction of expenditures); (g) South-South cooperation especially with Asian countries for health workforce development.
Strengthening of health systems	Regionalization of health centres

**Figure 2:**
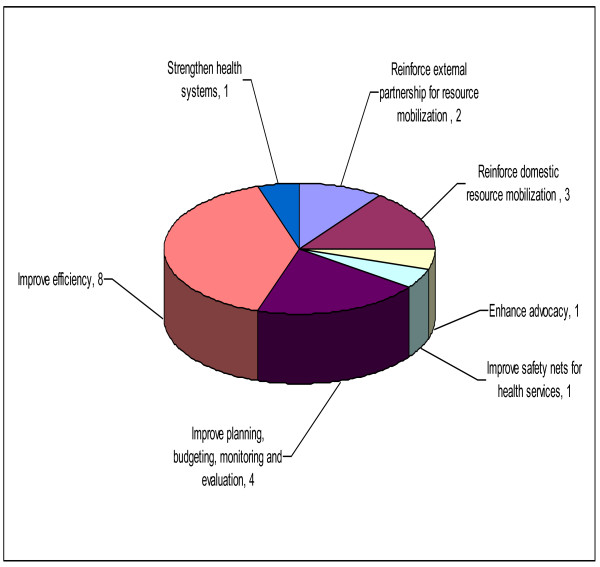
**Measures national authorities should take to mitigate negative effects of possible reduction in funding for health**.

Seventeen (89%) countries responded to the question seeking the respondents' opinion on other measures they thought that national authorities should take to mitigate any negative effects of possible reductions in funding for health development. Figure 2 provides a summary of the responses. In the opinion of the individuals who completed the questionnaire, the four "other" strategies most frequently proposed were improving efficiency (76.5%); reinforcement of domestic resource mobilization (41.2%); reinforcement of external partnership for resource mobilization (17.6%); and improving planning, budgeting, monitoring and evaluation (17.6%). Table 4 provides the broad strategies and specific measures that the respondents thought the national authorities should take to mitigate any negative effects of possible reduction in funding for health.

**Table 4:** Measures those national authorities should take to mitigate any negative effects of possible reduction in funding for health

Broad Strategies	Specific measures
Reinforce external partnership for resource mobilization	(a) Mobilize more external resources for health; (b) the government needs to be more proactive by working with donor/partners to estimate the possible effects of the global financial crisis achievement of the Millennium Development Goals; (c) the sector should engage global health initiatives to expand the scope of their funding to health systems development.
Reinforce domestic resource mobilization	(a) Explore other complementary financing mechanisms, e.g. social health insurance, community based health insurance; (b) households contributions through available schemes; (c) introduce standard fee for medication, while consultation remains free of charge at the point of delivery; (d) the need for government to improve upon tax revenue collection, especially major source of funding for National Health Insurance Fund is tax revenues, e.g. Ghana; (e) the sector should explore avenues for non traditional sources of funding by engaging more in public-private partnership at global and national levels; (f) request for banking credit to implement health priority health programmes that cannot be postponed.
Enhance advocacy	(a) Respect the Abuja Head of State commitment to allocate at least 15% of national budget to health; (b) advocacy for sustaining, if not increasing funding for health, which is necessary prerequisite for economic development.
Improve safety nets for health services	Continue to subsidize medicines
Improve planning, budgeting, monitoring and evaluation	(a). Money allocated to health should be available and utilized for health; (b) Monitor implementation of the poverty reduction strategy.
Improve efficiency	(a) Focus on priority public health interventions at primary level, including scaling up of health promotion and prevention; (b) reprioritize the use of funds; (c) reallocate resources from low priority sectors to high priority sectors like health; (d) focus on crucial health system inputs, e.g. health workforce, medicines, equipment maintenance; (e) halt new constructions or upgrades of infrastructure; (f) improve technical and allocative efficiency within the sector to ensure that the ministry gets value for value for all the limited resources spent; (g) pooled procurements.
Strengthen health facility management	Improve management capacities for health facilities at all levels

## Discussion

### Key findings

Three main responses fell within the aegis of other non-health sectors. Firstly, 83.3% (15/18) of respondents answered that price of basic food stuffs had increased recently. Secondly, 47.1% (8/17) reported increases in unemployment rates since the onset of the global financial crisis. And lastly, 38.8% (7/18) of the respondent countries recorded local currency devaluation vis-à-vis US dollar. These findings underscore the need for catalytic role of ministries of health in advocating for establishment of intersectoral mechanisms for mitigating effects of the global financial crisis on the social and economic determinants of health.

There were four key findings that fell within the scope of the ministry of health. Firstly, 37% (7/19) of respondent countries reported that the MOH had been notified by the MOF that budget for health will be cut as a result of the current global financial/economic crisis. Secondly, 15.8% (3/19) indicated that the MOH had been notified by partners of their intention to cut back their funding commitments. Thirdly, 61.1% (11/18) indicated that the prices of medicines had increased recently. Lastly, 64.7% (11/17) indicated that MOH had taken some measures (see Table 1 and Figure 1) already, either in reaction to the crisis, or in anticipation of it. Those measures are developed further in the section below.

### The Way Forward

The measures that some countries of the African Region are undertaking to mitigate negative effects of the global financial crisis on funding for health sector include: enhancing advocacy; improving efficiency; improving planning, budgeting, monitoring and evaluation; reinforcing domestic and external resource mobilization; improving safety nets for health services; and strengthening of health systems (see Figure 1). These actions are consistent with contents of the Framework for the implementation of the Ouagadougou Declaration on Primary Health Care and Health Systems in Africa [27].

#### Enhancing advocacy

Firstly, advocate for fiscal policy reform to allow for the allocation of more tax revenues for health development. Secondly, advocate nationally and through sub-regional economic communities for ministers of finance to sustain and increase domestic funding for the health sector in line with the Heads of State commitment to allocate at least 15% of national budgets to health [28]. This will require strengthening of capacities of ministries of health to dialogue with ministries of finance. Thirdly, advocate among development partners to fulfil their financial commitments to the health sector, including implementation of the Paris Declaration on Aid Effectiveness [29] and the Accra Agenda for Action [30].

#### Improving efficiency

There are a number of ways of improving efficiency - although not presented in any order. Firstly, institutionalize economic efficiency monitoring within national health management information systems with a view to implementing appropriate policy interventions to reduce wastage of scarce health system inputs [31]. Secondly, policy-makers can shift budgetary resources from low to high priority public health services/interventions, including health promotion and disease prevention. The process can be aided by available WHO cost-effectiveness evidence for choosing health interventions and programmes which maximize health from the available resources [32]. Lastly, improve management of medical supplies by developing transparent policies, procedures and criteria for medicine licensing, accreditation and approvals [17,18].

#### Improving planning, budgeting, monitoring and evaluation

It is critically necessary to reinforce ministries of health capacities for evidence-based planning, budgeting, monitoring, and evaluation. Firstly, reinforce the capacities for developing evidence-based costed national health sector strategic plans. Secondly, strengthen capacities within MOH for developing health component of the national Medium-Term Health Expenditure Framework (MTEF) and for public expenditure management [19].

Thirdly, institutionalize national health accounts to monitor the proportion of total government expenditure allocated to the health sector, household out-of-pocket spending on health as a percentage of total private health expenditure, and trends of external (donor) expenditure on health [33]. In the regional health financing strategy adopted by WHO Regional Committee for Africa in 2006, all 46 Ministers of Health agreed to institutionalize efficiency and equity monitoring and national and district health accounts within health information management systems. That regional policy agreement gives the Harmonization for Health in Africa (AfDB, JICA, UNAIDS, UNICEF, UNFPA, USAID, WHO, World Bank) and other relevant bilateral agencies a basis for collaborating with national authorities to develop a plan of action for rolling out support for institutionalizing national health accounts.

Finally, reinforce MOH capacities to monitor the effects of the economic crisis and policy responses on the social determinants of health (e.g. income level and distribution, unemployment, education, food, exchange rate fluctuations, volume of trade, tax revenues, government spending), health inputs (e.g. health workers, medicines, physical infrastructure, government and household expenditure on health, external health funding, financial policy linked to health), health system outputs (e.g. availability, prices, quality and efficiency of health services including prevention and promotion, utilization, risk behaviour), and health and health system outcomes [6,22].

#### Reinforcing domestic resource mobilization

The country respondents mentioned various ways of mobilizing more resources for health development. Firstly, develop financial risk-sharing mechanisms (e.g. social health insurance, community health insurance, private health insurance) through which to channel to households contributions which are currently paid directly to health service providers in form of out-of-pocket payments. Secondly, advocate at national cabinet forums for strengthening improvement in tax revenue collection and use of those revenues as especially a major source of funding for national health insurance fund, e.g., as in Ghana. Thirdly, explore non traditional sources of funding for health by engaging more in public-private partnerships at national level [34]. Lastly, depending on their context, countries may consider requesting for banking credit to implement health priority health programmes that cannot be postponed during the financial crisis. Some of the mechanisms proposed by the respondents are consistent with those recommended by the Taskforce on Innovative International Financing for Health Systems [35].

#### Reinforcing external resource mobilization

In order to attract more external resources for health, country respondents made a number of proposals. Firstly, the need to establish a partnership office in the MOH to identify and attract partners to co-fund the national health sector strategic plans. Secondly, proactively work with existing donor/partners to estimate the possible effects of the global financial crisis on the achievement of the health-related Millennium Development Goals. Thirdly, engage global health initiatives to expand the scope of their funding for health systems development [35].

#### Improving safety nets for health services

Countries that still have health service user fees should develop and implement effective exemption mechanisms to assure financial access for vulnerable groups [36]. In line with the Regional Committee resolution on health financing, countries should strengthen national prepaid health financing systems, including finance structures, processes and management systems [19]. This is necessary to ensure sharing of financial risk among the population and avoiding catastrophic health-care expenditure and impoverishment of care-seeking individuals.

#### Increase investments in national health systems

In line with the Ouagadougou Declaration on Primary Health Care and health systems in Africa, existing and additional funding from both national and international sources for the health sector needs to focus on overall systems strengthening including service delivery; health workforce; information; medicines, vaccines and technologies; financing; and leadership and governance [27]. This is the only way to optimize and sustain health gains, ensure responsiveness to client expectations and guarantee fairness in financial contributions to health service expenditures.

### Limitations

(a) The overall response rate of 41.3% (19/46) was low. It could be partially attributed to lack of involvement of the Ministries of Health (MOH) Directors of Policy and Planning in the development of the questionnaire. With hindsight, it would have been better to have taken time to involve them in identification of the variables for inclusion in the questionnaire, planning and implementing the survey. Therefore, while the responses provide useful information about the countries which responded, the response rate was too low to draw conclusions about how the region as a whole is reacting to the global financial crisis.

(b) Some of the questions may be best answered by officials in ministries other than those of health, e.g. the questions about local currency devaluation, unemployment and food prices. Unfortunately, we are not sure whether the respondents consulted other relevant Ministries (e.g. Agriculture, Finance and Labour) in the process of completing the survey questionnaire.

(c) Since it was a quick survey, we missed the opportunity to explore/probe why there was a big difference between what respondents reported has been done to mitigate the effects of the global financial crisis on health sector funding and development, and what they indicated *could *be done. We agree with peer reviewers that exploring why countries may have a good understanding of what could be done but have not done it would be an important first step in identifying what should be done to bridge the "know-do gap".

### Suggestions for future surveys

(a) Prior to further use, the existing questionnaire should be revised through active involvement of Directors of Policy and Planning in Ministries of Health, and their counterparts in other relevant ministries.

(b) The non-health questions should be administered by Directors of Policy and Planning in MOH to their counterparts in other relevant ministries.

(c) Those administering the questionnaire should probe: (i) why there may be a "know-do gap"; (ii) whether there are trade-offs that affect policy choices of taking specific actions to mitigate financial constraints, e.g. whether efforts to mobilize external resources raises trade-offs with areas of economic efficiency by concentrating resources in areas preferred by external financiers; and (iii) how to promote the use of national health accounts for purposes of monitoring the effect of the global financial crisis on the health sector.

## Conclusion

A rapid assessment, like the one reported in this article, of the effects of the global financial crisis on a few variables is important to alert the Ministry of Health on the looming dangers of cuts in health funding from domestic and external sources. However, it is even more important for national governments to monitor the effects of the economic crisis and policy responses on the social determinants of health, health inputs, health system outputs, and health outcomes [6]. The current situation where developed countries are struggling to address their own budget deficits underscores the need for African countries to use all funding efficiently and to continue monitoring the financial situation and taking appropriate measures to protect funding to the health sector.

## List of abbreviations

AfDB: African Development Bank; GDP: Gross domestic product; IMF: International Monetary Fund; JICA: Japanese International Cooperation Agency; MOF: Ministry of Finance; MOH: Ministry of Health; MTEF: Medium-Term Health Expenditure Framework; NHA: National Health Accounts; ODA: Official Development Assistance; RC: WHO Regional Committee for Africa; UNAIDS: Joint United Nations Programme on HIV/AIDS; UNFPA: United Nations Population Fund; UNICEF: United Nations Children's Fund; USAID: United States Agency for International Development; WHO: World Health Organization; WHO/AFRO: World Health Organization Regional Office for Africa;

## Competing interests

The authors declare that they have no competing interests.

## Authors' contributions

JMK, BMN, CNM and BC contributed to the study design, analysis and writing of various sections of the manuscript. All authors read and approved the final manuscript.

## Pre-publication history

The pre-publication history for this paper can be accessed here:

http://www.biomedcentral.com/1472-698X/11/4/prepub

## Supplementary Material

Additional file 1 **Continuous monitoring of the effects of global financial crisis on funding for health development: a questionnaire for completion by directors of policy and planning at the Ministry of Health**. Click here for file
