# Supplementary material for: Effects of global financial crisis on funding for health development in nineteen countries of the WHO African Region
Source: BMC Int Health Hum Rights. 2011 Apr 13;11:4. doi: 10.1186/1472-698X-11-4 (PMC3094291; doi:10.1186/1472-698X-11-4)
Supplement: Additional file 1 — Continuous monitoring of the effects of global financial crisis on funding for health development: a questionnaire for completion by directors of policy and planning at the Ministry of Health. [file 1472-698X-11-4-S1.DOC]

**CONTINUOUS MONITORING OF THE EFFECTS OF GLOBAL FINANCIAL CRISIS ON FUNDING FOR HEALTH DEVELOPMENT: a questionnaire for completion by Directors of Policy and Planning at the Ministry of Health**

**Respondent contacts:**

**Country:______________________________________________________________**

**Name & position of respondent :__________________________________________**

**Telephone number of respondent:_________________________________________**

**Email address of respondent: _____________________________________________**

**Preamble**

The developed countries are already in a recession, i.e. a situation of declining gross domestic product (GDP), or negative real economic growth, lasting for two or more successive quarters of a year or are nearly into one. There are concerns that the world is heading for a global recession. A recession has many attributes that can occur simultaneously and can include declines in coincident measures of activity such as employment, incomes, investment, corporate profits and government tax revenues and expenditures.

There is a real danger that funding for health development in the African Region might be adversely affected by the ongoing global economic/financial crisis. The current crisis threatens to derail the ongoing national and international efforts to realize the Millennium Development Goals in many countries. Therefore, there is need for concerted action from African Governments and development partners to ensure that domestic and external funding for the health sector is not reduced.

Also there is need to vigilantly monitor and report regularly any official and unofficial expressions of intentions by ministries of finance or partners to change the current or future levels of funding for health development in your country of duty. WHO is currently working with countries to monitor the effects of the global economic/financial crisis on funding for health development. The purpose of monitoring is to ensure that prompt action is taken to advocate with national authorities and partners whenever there are indications of reductions in funding for health.

In order to facilitate continuous monitoring of effects of the economic crisis, kindly forward to WHO Country Representative answers to the questions below every month.

**Questions**

1. Has the Ministry of Health been notified that the budget for health will be cut as a result of the current global financial/economic crisis?

(i) Yes (ii) No (iii) Don’t know.

1. If yes in question (a), do they know if, at the same time, the share of total government spending going to health will:

(i) Increase (ii) Fall (iii) Stay the same (iv) Do not know.

1. Has any development Partner (donor) notified the Ministry of Health that their funding commitments will be cut as a result of the current global financial/economic crisis?

(i) Yes (ii) No (iii) Don’t know.

1. If Yes in question (c), please indicate the names of the development partners and the amounts by which their funding for health is likely to decrease.

________________________________________________________________________________________________________________________________________

1. Is there any indication that the prices of medicines have increased recently?

(i) Yes (ii) No (iii) Do not know

1. If Yes in question (e), is there any information available about how much the prices have increased?

________________________________________________________________________________________________________________________________________

1. Is there any indication that the prices of basic food stuffs have increased recently?

(i) Yes (ii) No (iii) Do not know

1. Has the local currency been devalued since the global financial crisis?

(i) Yes (ii) No (iii) Do not know

1. If Yes in question (h), by what percentage? _________________________________
2. Have the levels of unemployment increased since the global financial crisis?

(i) Yes (ii) No (iii) Do not know

1. If Yes in question (j), what are the reasons for increasing unemployment?

____________________________________________________________________________________________________________________________________________________________________________________________________________________________________________________________________________________________________________________________________________________

1. Has the Ministry of Health taken any policy measures already, either in reaction to the crisis, or in anticipation?

(i) Yes (ii) No.

1. If Yes in question (l), kindly indicate below what measures the Ministry of health has already taken to mitigate any negative effects of possible reduction in funding for health.

____________________________________________________________________________________________________________________________________________________________________________________________________________________________________________________________________________________________________________________________________________________

1. In your opinion, what other measures do you think that national authorities should take to mitigate any negative effects of possible reductions in funding for health development?

____________________________________________________________________________________________________________________________________________________________________________________________________________________________________________________________________________________________________________________________________________________________________________________________________________________________________________________________________________________________

**THANK YOU VERY MUCH FOR YOUR COOPERATION AND TIME**
